# ECACS: An Enhanced Certificateless Authentication Scheme for Smart Car Sharing

**DOI:** 10.3390/s25175441

**Published:** 2025-09-02

**Authors:** Zhuowei Shen, Xiao Kou, Taiyao Yang

**Affiliations:** 1 School of Cyberscience and Engineering, Southeast University, Nanjing 211189, China; 220235211@seu.edu.cn (X.K.); 220235271@seu.edu.cn (T.Y.); 2 Key Laboratory of Computer Network and Information Intergration, Minster of Education, Nanjing 211189, China

**Keywords:** car-sharing, certificateless signature, internet of vehicles, authentication

## Abstract

Driven by the demand for cost-effective vehicle access, enhanced flexibility, and sustainable transportation practices, smart car-sharing has emerged as a prominent alternative to traditional vehicle rental systems. Leveraging the Internet of Vehicles (IoV) and wireless communication, these systems feature dynamic renter-vehicle mappings, enabling users to access any available vehicle rather than being restricted to a specific one pre-assigned by the service provider. However, many existing schemes in the IoV field conflate users and vehicles, complicating the identification and tracking of the vehicle’s actual driver. Moreover, most current authentication protocols rely on a strict, initial binding between a user and a vehicle, rendering them unsuitable for the dynamic nature of car-sharing environments. To address these challenges, we propose an enhanced certificateless signature scheme tailored for smart car-sharing. By employing a biometric fuzzy extractor and the Chinese Remainder Theorem, our scheme provides a fine-grained authentication mechanism that eliminates the need for local computations on the user’s side, meaning users do not require a smartphone or other digital device. Furthermore, our scheme introduces category identifiers to facilitate vehicle selection based on specific classes within car-sharing contexts. A formal security analysis demonstrates that our scheme is existentially unforgeable against adversaries under the random oracle model. Finally, a comprehensive evaluation shows that our proposed scheme achieves competitive performance in terms of computational and communication overhead while offering enhanced practical functionalities.

## 1. Introduction

The widespread adoption of digital technologies and the development of the Internet of Vehicles (IoV) are driving the transformation of the automotive sector toward intelligent systems [1]. For instance, connected vehicles exchange real-time traffic information through vehicle-to-vehicle communication, while smart parking systems leverage positional data to optimize space allocation. Furthermore, within the framework of intelligent transportation systems, all automotive entities can contribute to optimizing traffic flow and enhancing overall transportation efficiency through global monitoring and data analytics [2,3]. In recent years, driven by the growing demand for cost-effective mobility, smart car-sharing has gained significant traction as an alternative to traditional vehicle rental services [4]. According to a report by Statista Market Insights, global car-sharing revenue is projected to reach approximately USD 13.89 billion in 2025 and is forecast to grow at a compound annual growth rate (CAGR) of 3.23% over the subsequent five-year period [5].

**Figure 1:**
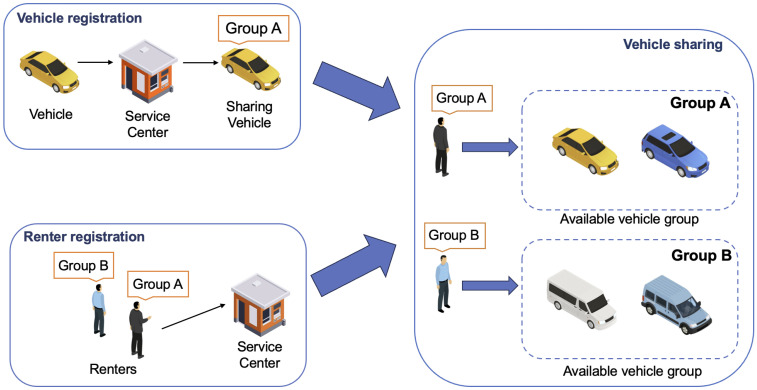
A general scenario of smart car-sharing.

Traditional car rental services require users to book a specific vehicle in advance and retrieve it from a designated depot, a process that is often time-consuming and inconvenient. In contrast, smart car-sharing providers (e.g., Zipcar, Car2Go) offer a more flexible alternative. As illustrated in Figure 1, a typical smart car-sharing workflow begins with vehicles being classified into categories based on their characteristics (e.g., model, capacity) and registered with the service center. After completing registration and payment, users can select any available vehicle within a category that meets their requirements. This model allows individuals to use vehicles on demand, thereby avoiding the costs and responsibilities associated with private car ownership, such as maintenance expenses [6]. Furthermore, compared with public transportation and conventional car rentals, smart car-sharing provides greater flexibility in travel time and destination, as well as more convenient vehicle access [7].

Despite these benefits, communication in IoV environments relies on wireless channels, which are inherently insecure and susceptible to malicious attacks. Consequently, various authentication schemes [8,9,10] have been proposed for the IoV domain to safeguard against threats such as replay [11] and forgery attacks [12]. However, a prevalent limitation of these protocols is their static binding of users to vehicles, which effectively treats the pair as an inseparable entity. This approach is ill-suited for dynamic car-sharing environments. For instance, when malicious behavior occurs, traditional schemes can typically only identify the specific vehicle involved, not the driver. As a result, preventing the malicious user from accessing other vehicles is a challenging and delayed process, as existing authentication protocols incorporate vehicle-specific, rather than user-specific, information in their computations. In a system characterized by rapid user turnover, this oversight creates a significant security vulnerability.

Furthermore, the high turnover of renters for a single vehicle necessitates schemes with dynamic membership management capabilities. Various approaches have been proposed to achieve this functionality, including the use of area keys [13] and Merkle Hash Trees (MHTs) [14]. However, the former approach is computationally expensive due to the need for individual calculations per user, while the latter incurs significant storage overhead because of the hierarchical structure of hash trees. In contrast, the Chinese Remainder Theorem (CRT) enables efficient and lightweight membership management by maintaining and distributing a global shared secret value through broadcasting, demonstrating advantages in both computational efficiency and storage overhead [15]. Nevertheless, current applications of CRT in the IoV field primarily focus on vehicle-side applications. These implementations often require users to perform computations on their personal digital devices, such as smartphones, which can be impractical or inconvenient for renters in real-world scenarios.

In response to the aforementioned challenges, we propose an enhanced CLS scheme that introduces a fine-grained and user-vehicle decoupled authentication mechanism for smart car-sharing scenarios. Specifically, our scheme employs the fuzzy extractor to securely incorporate user biometric information into the authentication process while eliminating the need for user-side computation. Once a user completes the login procedure, the corresponding vehicle generates a signature embedded with a biometric-derived secret, thereby enhancing authenticity by jointly verifying both the user and the vehicle. Additionally, vehicles within the system are classified into categories, with each assigned a unique identifier. This process enables legitimate users to select and access any available vehicle within their chosen category. Furthermore, we integrate the CRT with user-extracted biometric secrets and facilitate the dynamic revocation of renters in cases of service expiration or malicious behavior. The key contributions of the article are outlined as follows:

(1) We propose an efficient CLS authentication scheme for smart car-sharing scenarios, built upon the fuzzy extractor and the CRT. Compared with existing authentication schemes, our scheme provides decoupled, fine-grained authentication, supports dynamic user revocation, and eliminates computation on the user side.
(2) We introduce a novel category-range authentication framework where vehicles are organized into distinct categories with unique identifiers. This enables users to access any vehicle within an authorized category, instead of a single specific one in most IoV frameworks, thereby enhancing the flexibility and efficiency of the rental process.
(3) We conduct a comprehensive security analysis of our scheme and benchmark its performance against related works. The results demonstrate that our proposal achieves provable security and exhibits competitive overhead in terms of computation, communication, and dynamic membership management.

The remainder of this article is structured as follows: Section 2 reviews the existing related works relevant to our proposed scheme. Section 3 introduces the preliminaries, as well as our system and security models. Section 4 elaborates on the details of our proposed scheme. Subsequently, Section 5 presents the formal security analysis and discusses the security properties of our scheme. In Section 6, we evaluate the performance of our proposed scheme and compare it with other existing schemes. Finally, Section 7 concludes the article.

## 2. Related Works

Digital signature schemes have been widely adopted in the IoV to secure communication processes. In early approaches based on Public Key Infrastructure (PKI) [16,17], certificates and the corresponding public–private key pairs of the vehicles are issued by a trusted certificate authority (CA). Although the implementation of PKI systems enhances the security level of these schemes, the resource-intensive certificate management process remains a significant concern. To mitigate this issue, subsequent protocols leveraged Identity-Based Cryptography (IBC), which eliminates certificate management overhead by employing a trusted Private Key Generator (PKG) to derive public keys directly from identities [18,19,20]. However, the implementation of PKG also introduces the key escrow problem, which can lead to sensitive information exposure and faulty authentication if the PKG in the scheme is compromised and no longer trusted.

To address the key escrow problem, Al-Riyami and Paterson proposed the concept of certificateless public key cryptography (CL-PKC) [21]. By replacing PKG with a semi-trusted key generation center (KGC) and incorporating random numbers selected by users themselves in the key generation process, certificateless signature (CLS) schemes achieve private key confidentiality, as KGC cannot acquire the complete private key of the participants [22].

Built on the framework of CL-PKC, numerous CLS schemes with varying characteristics have been proposed. For instance, Malip et al. [23] proposed a certificateless anonymous authentication scheme designed to facilitate secure message transmission. Zhong et al. [24] introduced a certificateless aggregate signature scheme (CLAS) incorporating pseudonyms to achieve message authentication and conditional privacy preservation. Wang et al. [25] proposed a novel certificateless authentication scheme and demonstrated its security within the standard model. However, the scheme proposed by Wang et al. was subsequently proven to be insecure [26]. Furthermore, the aforementioned schemes are all based on bilinear mapping, which is computationally intensive, raising significant concerns regarding their efficiency.

To reduce the computational overhead associated with bilinear pairings, schemes based on elliptic curve cryptography (ECC) have been widely proposed [27,28,29,30,31,32]. Ali et al. [33] proposed a certificateless authentication scheme utilizing map-to-point hash functions and short signatures. However, Li et al. [34] later identified that the scheme proposed in [33] is vulnerable against Type-II adversaries and subsequently contributed a new reliable CLAS scheme for VANET. Aiming to solve the weakness of the potential side channel attacks against tamper-proof devices, Xiong et al. [35] proposed a double insurance CLS scheme. Unfortunately, Shim [26] later demonstrated that Xiong’s scheme is susceptible to forgery attacks. Following this, Gong et al. [36] introduced a certificateless authentication protocol, asserting its robustness against both Type-I and Type-II adversaries while claiming superiority over prior schemes in [35,37]. Nevertheless, Wu et al. [38] recently concluded that the scheme by Gong et al. [36] also fails to achieve the claimed resistance against forgery attacks by Type-I adversaries. Crucially, in IoV-based car-sharing scenarios, these schemes focus solely on inter-vehicle authentication while neglecting the essential granular authentications required between users and vehicles, thereby rendering them impractical for dynamic car-sharing applications.

In recent years, numerous schemes have sought to enhance the flexibility of users in IoV by incorporating techniques such as passwords and biometric information [39,40]. Islam et al. [41] proposed a password-based authentication protocol for VANETs that supports the dynamic changes in vehicle membership. Later, Cui et al. [42] introduced an authentication scheme based on the Chebyshev chaotic map, incorporating fuzzy verification and honeywords for rescue scenarios. Regarding the application of biometric information, Azees et al. [43] proposed a hybrid CMOS memristor-based authentication scheme employing bilinear operations, ensuring that only registered users can interact with the vehicle. Wang et al. [44] integrated distance calculation into the biometric authentication process and proposed a fuzzy CLS scheme. Despite these advancements, existing approaches maintain a rigid binding between users and vehicles, preventing users from accessing vehicles beyond their specifically assigned ones. Meanwhile, in smart car-sharing scenarios, it is crucial for service providers to allow renters to access vehicles at their convenience.

A comparative summary of existing schemes is presented in Table 1. The limitations identified therein highlight the need for a practical authentication scheme that decouples users from specific vehicles, thereby better addressing the operational requirements of real-world car-sharing services.

## 3. Preliminaries

In this section, we first provide a concise background explanation of elliptic curve cryptography and the Chinese Remainder Theorem. Then, we introduce the system and security models of our proposed authentication scheme. The symbols used in our scheme are described in Table 2.

### 3.1. Elliptic Curve Cryptography

In the ECC system, an elliptic curve *E* is defined by the equation $y^{2}=x^{3}+ax+b\;\mathrm{mod}\;p$ over $F_{p}$, where $F_{p}$ is a finite field defined on a large prime number *p*. Here, $a,b\in F_{p}$, satisfying the condition $4a^{3}+27b^{2}\;\mathrm{mod}\;p\neq 0$. Furthermore, let *O* represent a point at infinity; then an additive elliptic curve group G with order *q* and the generator *P* can be defined with *O* and the points on *E*.

Elliptic Curve Discrete Logarithm Problem (ECDLP): Given the instance (P,aP), where $a\in Z_{q}^{*}$ and P∈G, it is computationally infeasible to obtain *a* from aP in probabilistic polynomial time.

### 3.2. Chinese Remainder Theorem

Let $m_{1},m_{2},\cdots ,m_{n}$ be pairwise coprime positive integers, and let $a_{1},a_{2},\cdots ,a_{n}$ denote arbitrary integers. Then, the system

$$\left\{\begin{array}{c} x\equiv a_{1}\;\left(\mathrm{mod}\;m_{1}\right)\\ x\equiv a_{2}\;\left(\mathrm{mod}\;m_{2}\right)\\ \cdots \\ x\equiv a_{n}\;\left(\mathrm{mod}\;m_{n}\right) \end{array}\right.$$

has a unique solution modulo $N=\prod_{i=1}^{n}m_{i}$. Specifically, the solution can be expressed as $x\equiv \sum_{i=1}^{n}a_{i}\times \alpha_{i}\times \beta_{i}\;\mathrm{mod}\;N$, where $\alpha_{i}=\frac{N}{m_{i}}$ and $\alpha_{i}\times \beta_{i}\equiv 1\;\mathrm{mod}\;m_{i}$.

### 3.3. System Model

In our proposed scheme, four main participants are involved, namely the TA (trust authority), the KGC (key generation center), the SV (sharing vehicle), and the users.

1. TA: It represents the service center in smart car-sharing, which is a fully trusted authority that generates the system parameters and manages the service membership of users within the system by distributing and updating the group identifier. Moreover, it is also responsible for generating pseudonyms for users and can track and reveal their real identities when malicious behaviors occur.
2. KGC: It represents a key generation center that generates the partial private keys for vehicles and the private keys for users and is considered to be a semi-trusted third party.
3. SV: It represents a vehicle in the car-sharing service. In our scheme, SVs are classified into individual categories based on their characteristics (e.g., model and capacity). After an SV is logged in by a legitimate renter via biometric information, it can authenticate with other verified SVs by generating signatures utilizing the derived group identifier.
4. User: It represents the actual user of the car-sharing service. Additionally, it is important to emphasize that no local computational abilities are required for users in our proposed scheme.

**Figure 2:**
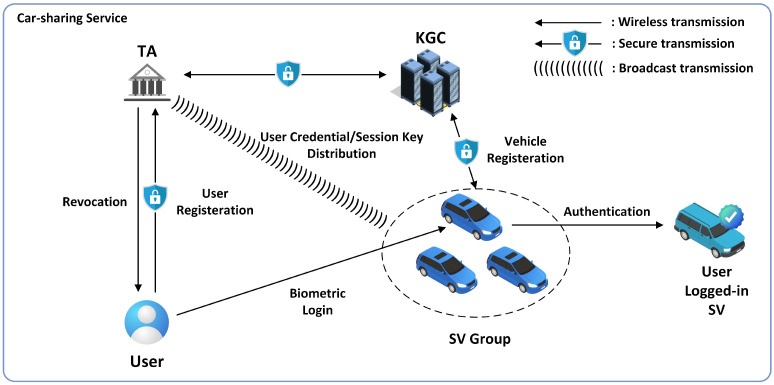
The system model of the proposed scheme.

The system model of our proposed scheme is demonstrated in Figure 2. Initially, SVs register with KGC to obtain their partial private keys and category identifiers. Subsequently, users complete the registration process by submitting biometric information to TA. When a legitimate user requires access to a vehicle, they may access any vehicle that shares the same category identifier selected during user registration. Upon successful user login, the vehicle can generate signatures to enable secure authentication with other accessed vehicles. In the event that a malicious user is detected, TA will update the group identifier and broadcast the auxiliary information to process revocation, and legitimate vehicles can obtain the updated group identifier using their identifier update secrets derived from the users.

### 3.4. Security Model

In the field of CLS schemes, two types of attacks are often considered, namely Type-I attacks launched by external adversaries and Type-II attacks launched by internal adversaries [45]. In a Type-I attack, an adversary is able to launch public key replacement attacks but cannot gain access to the master secret key. In contrast, in a Type-II attack, an adversary can acquire the system’s master secret key but cannot replace the public key of any entity.

To demonstrate the security of our proposed scheme, we will prove its existential unforgeability against chosen-message attacks (EUF-CMA). This proof is based on two challenge-response games, each formalizing one of the aforementioned attack scenarios.

Game I: This game is performed by a Type-I adversary $A_{1}$ and the challenger C. The details are as follows:

Setup: C runs Setup to generate its master secret key *s* and the system parameters params. Then it sends params to $A_{1}$ and keeps *s* secret.

Queries: In this phase, $A_{1}$ can adaptively access the following queries.

(1) Vehicle Registration Query: When receiving this query from $A_{1}$ with ${\mathrm{PVID}}_{i}$ as input, C generates the related information for the corresponding vehicle.
(2) User Registration Query: When receiving this query from $A_{1}$ with ${\mathrm{PID}}_{j}$ as input, C generates the related information for the corresponding user.
(3) Reveal Vehicle Partial Key Query: When receiving this query from $A_{1}$ with ${\mathrm{PVID}}_{i}$ as input, C returns the corresponding $d_{i}$.
(4) Reveal User Private Key Query: When receiving this query from $A_{1}$ with ${\mathrm{PID}}_{j}$ as input, C returns the corresponding $d_{j}$.
(5) Reveal Vehicle Secret Query: When receiving this query from $A_{1}$ with ${\mathrm{PVID}}_{i}$ as input, C returns the corresponding $x_{i}$.
(6) Reveal Vehicle Public Key Query: When receiving this query from $A_{1}$ with ${\mathrm{PVID}}_{j}$ as input, C returns the corresponding $\left(X_{i},R_{i}\right)$.
(7) Reveal User Public Key Query: When receiving this query from $A_{1}$ with ${\mathrm{PID}}_{j}$ as input, C returns the corresponding $R_{j}$.
(8) Replace Vehicle Public Key Query: When receiving this query from $A_{1}$ with $\left({\mathrm{PVID}}_{i},X_{i}^{\prime },R_{i}^{\prime }\right)$ as input, C replaces the public key of the corresponding vehicle with $X_{i}^{\prime },R_{i}^{\prime }$.
(9) Replace User Public Key Query: When receiving this query from $A_{1}$ with $\left({\mathrm{PID}}_{j},R_{j}^{\prime }\right)$ as input, C replaces the public key of the corresponding user with $R_{j}^{\prime }$.
(10) Sign Query: When receiving this query from $A_{1}$ with $\left({\mathrm{PVID}}_{i},{\mathrm{PID}}_{j},m_{i}\right)$ as input, C outputs a legitimate signature $\sigma_{i}$.

Forgery: In this phase, $A_{1}$ outputs a signature $\sigma^{*}$ for the tuple $\left({\mathrm{PVID}}_{i},{\mathrm{PID}}^{*},m^{*}\right)$, and $A_{1}$ wins in this game if the following conditions are satisfied.

- Sign Query has never been queried with the submitted tuple.
- Reveal User Private Key Query and Replace User Public Key Query have never been queried with the pseudonym ${\mathrm{PID}}^{*}$ by $A_{1}$.
- $\sigma^{*}$ is a valid signature for the submitted tuple.

Game II: This game is performed by a Type-II adversary $A_{2}$ and the challenger C. The details are as follows:

Setup: C runs Setup to generate its master secret key *s* and the system parameters params. Then it sends params and *s* to $A_{2}$.

Queries: In this phase, $A_{2}$ can adaptively access the following queries.

(1) Vehicle Registration Query: When receiving this query from $A_{2}$ with ${\mathrm{PVID}}_{i}$ as input, C generates the related information for the corresponding vehicle.
(2) User Registration Query: When receiving this query from $A_{2}$ with ${\mathrm{PID}}_{j}$ as input, C generates the related information for the corresponding user.
(3) Reveal User Private Key Query: When receiving this query from $A_{2}$ with ${\mathrm{PID}}_{j}$ as input, C returns the corresponding $d_{j}$.
(4) Reveal Vehicle Secret Query: When receiving this query from $A_{2}$ with ${\mathrm{PVID}}_{i}$ as input, C returns the corresponding $x_{i}$.
(5) Reveal Vehicle Public Key Query: When receiving this query from $A_{2}$ with ${\mathrm{PVID}}_{j}$ as input, C returns the corresponding $\left(X_{i},R_{i}\right)$.
(6) Reveal User Public Key Query: When receiving this query from $A_{2}$ with ${\mathrm{PID}}_{j}$ as input, C returns the corresponding $R_{j}$.
(7) Sign Query: When receiving this query from $A_{2}$ with $\left({\mathrm{PVID}}_{i},{\mathrm{PID}}_{j},m_{i}\right)$ as input, C outputs a legitimate signature $\sigma_{i}$.

Forgery: In this phase, $A_{2}$ outputs a signature $\sigma^{*}$ for the tuple $\left({\mathrm{PVID}}^{*},{\mathrm{PID}}_{j},m^{*}\right)$, and $A_{2}$ wins in this game if the following conditions are satisfied.

- Sign Query has never been queried with the submitted tuple.
- Reveal Vehicle Secret Query has never been queried with the pseudonym ${\mathrm{PVID}}^{*}$ by $A_{2}$.
- $\sigma^{*}$ is a valid signature for the submitted tuple.

## 4. Proposed Scheme

In this section, we describe our proposed scheme in detail.

### 4.1. Setup

First, TA and KGC will initialize the system by generating public and secret parameters.

(1) Given λ as the security parameter, TA chooses a cyclic group G of order *q* with *P* as its generator. Then, TA randomly selects $t\in Z_{q}^{*}$ and calculates $T_{pub}=t\cdot P$.
(2) Then, TA selects five secure hash functions: $H_{0}:G\times {\left\{0,1\right\}}^{*}\to Z_{q}^{*}$, $H_{1}:{\left\{0,1\right\}}^{*}\times G^{3}\to Z_{q}^{*}$, $H_{2}:{\left\{0,1\right\}}^{*}\times G^{2}\times {\left\{0,1\right\}}^{*}\to Z_{q}^{*}$, $H_{3}:{\left\{0,1\right\}}^{*}\times {\left\{0,1\right\}}^{*}\times G^{4}\times {\left\{0,1\right\}}^{*}\times {\left\{0,1\right\}}^{*}\to Z_{q}^{*}$, $H_{4}:{\left\{0,1\right\}}^{*}\times {\left\{0,1\right\}}^{*}\times G^{2}\times {\left\{0,1\right\}}^{*}\times {\left\{0,1\right\}}^{*}\to Z_{q}^{*}$.
(3) KGC randomly selects $s\in Z_{q}^{*}$ and calculates $P_{pub}=s\cdot P$.
(4) TA selects *n* unique prime numbers ${Prime}_{k=1,2,\cdots ,n}$ and calculates $\phi_{g}=\prod_{k=1}^{n}Prime_{k}$. Then, for each ${Prime}_{k}$, TA calculates $\alpha_{k}=\frac{\phi_{g}}{{Prime}_{k}}$, $\alpha_{k}\times \beta_{k}\equiv 1\;\mathrm{mod}\;Prime_{k}$, $\rho_{k}=\alpha_{k}\times \beta_{k}$ individually.
(5) Finally, TA calculates $\Omega =\sum_{k=1}^{n}\rho_{k}$ and publishes the system parameters, which are denoted as $params=\{G,q,P,P_{pub},T_{pub},H_{0},H_{1},H_{2},H_{3},H_{4},\Omega \}$.

### 4.2. Registration

Every entity, both the SVs and the users, will first complete the registration after system initialization. The registration process includes vehicle registration, user registration, and group identifier distribution.

#### 4.2.1. Vehicle Registration

**Figure 3:**
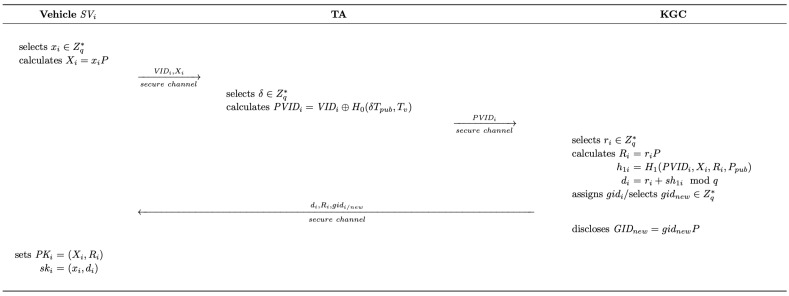
The vehicle registration phase of the proposed scheme.

As demonstrated in Figure 3, when an SV proceeds with registration, it first generates its partial key and transmits its unique identity to KGC. Upon receiving the request, KGC generates the corresponding partial private key and assigns a category identifier to the SV. Later, according to the received information from KGC, SV can complete its public–private key pair.

(1) A vehicle ${\mathrm{SV}}_{i}$ first randomly selects $x_{i}\in Z_{q}^{*}$ and calculates $X_{i}=x_{i}P$. Then, it sends $\left\{{\mathrm{VID}}_{i},X_{i}\right\}$ to TA through a secure channel, where ${\mathrm{VID}}_{i}$ denotes the unique identifier of ${\mathrm{SV}}_{i}$.
(2) Upon receiving the request from ${\mathrm{SV}}_{i}$, TA randomly selects $\delta \in Z_{q}^{*}$ and calculates ${\mathrm{PVID}}_{i}={\mathrm{VID}}_{i}⊕H_{0}\left(\delta T_{pub},T_{v}\right)$, where $T_{v}$ is the current timestamp. Consequently, TA transmits ${\mathrm{PVID}}_{i}$ to KGC through a secure channel.
(3) After receiving information from TA, KGC randomly selects $r_{i}\in Z_{q}^{*}$ and calculates $R_{i}=r_{i}P$, $h_{1i}=H_{1}\left({\mathrm{PVID}}_{i},X_{i},R_{i},P_{pub}\right)$, $d_{i}=r_{i}+sh_{1i}\;\mathrm{mod}\;q$. Then, based on the characteristics of the vehicle (e.g., model, capacity), KGC assigns an existing or creates a new class identifier $gid_{i}\in Z_{q}^{*}$ to ${\mathrm{SV}}_{i}$ and transmits $\left\{d_{i},R_{i},gid_{i}\right\}$ to ${\mathrm{SV}}_{i}$ through a secure channel. Note that every time after a new class identifier $gid_{new}$ is created and assigned, KGC calculates $GID_{new}=gid_{new}P$ and discloses it to all the entities within the system.
(4) After obtaining the message from KGC, ${\mathrm{SV}}_{i}$ sets its public key as ${\mathrm{PK}}_{i}=\left(X_{i},R_{i}\right)$ and its private key as $sk_{i}=\left(x_{i},d_{i}\right)$.

#### 4.2.2. User Registration

**Figure 4:**
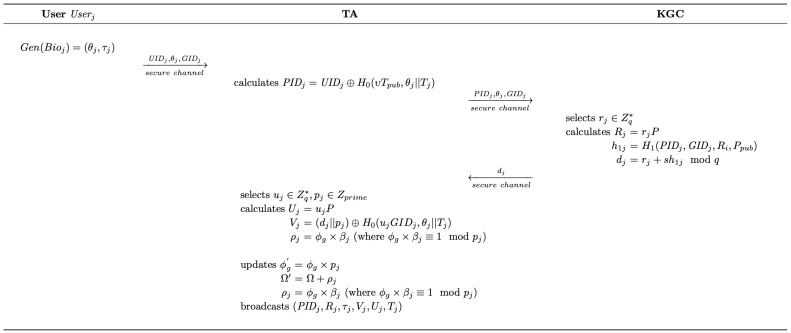
The user registration phase of the proposed scheme.

As presented in Figure 4, when registering, users are required to provide their biometric information. Then, based on the pre-selected category identifier and the pseudonym generated, KGC generates the corresponding credentials, which are consequently encrypted and broadcast to all SVs within the system by TA.

(1) With the real user identity ${\mathrm{UID}}_{j}$, the target category identifier $GID_{j}$, and the biometric information $Bio_{j}$ acquired from ${\mathrm{User}}_{j}$, TA first utilizes the fuzzy extractor to calculate $Gen\left(Bio_{j}\right)=\left(\theta_{j},\tau_{j}\right)$. Then, TA randomly selects $\upsilon \in Z_{q}^{*}$ and calculates ${\mathrm{PID}}_{j}={\mathrm{UID}}_{j}⊕H_{0}(\upsilon T_{pub},\theta_{j}\left|\right|T_{j})$, where $T_{j}$ is the current timestamp. Consequently, TA transmits $\left\{{\mathrm{PID}}_{j},\theta_{j},GID_{j}\right\}$ to KGC through a secure channel.
(2) Upon receiving the data of TA, KGC randomly selects $r_{j}\in Z_{q}^{*}$ and calculates $R_{j}=r_{j}P$. Then, KGC calculates the credential $d_{j}=r_{j}+sh_{1j}\;\mathrm{mod}\;q$ for ${\mathrm{User}}_{j}$ and transmits it to TA via a secure channel, where $h_{1j}=H_{1}\left({\mathrm{PID}}_{j},GID_{j},R_{j},P_{pub}\right)$.
(3) Next, TA randomly selects $u_{j}\in Z_{q}^{*}$ and an identifier update secret $p_{j}\in Z_{prime}$. Then, TA calculates $U_{j}=u_{j}P$ and $V_{j}=(d_{j}\left|\right|p_{j})\;⊕\;H_{0}(u_{j}GID_{j},\theta_{j}\left|\right|T_{j})$.
(4) TA calculates $\phi_{g}\times \beta_{j}\equiv 1\;\mathrm{mod}\;p_{j}$, $\rho_{j}=\phi_{g}\times \beta_{j}$ and records the parameters in a list *L*.
(5) Finally, TA updates $\phi_{g}$ with $\phi_{g}^{\prime }=\phi_{g}\times p_{j}$, Ω with $\Omega^{\prime }=\Omega +\rho_{j}$ and broadcasts $\left({\mathrm{PID}}_{j},R_{j},\tau_{j},V_{j},U_{j},T_{j}\right)$ through a public channel.

#### 4.2.3. Group Identifier Distribution

To transmit the latest group identifier to all SVs, TA first randomly selects $w,z\in Z_{q}^{*}$ and calculates W=wP, ζ=zΩ, and $\xi =w+H_{2}\left(\zeta ,W,T_{pub},T_{z}\right)t$, where $T_{z}$ denotes the current timestamp. Later, TA broadcasts $\left(\zeta ,W,\xi ,T_{z}\right)$ to all vehicles within the system.

Upon receiving the message from TA, SVs will first check the freshness of the message. Then, they will verify the authenticity of ζ by checking whether the equation $\xi P=W+H_{2}\left(\zeta ,W,T_{pub},T_{z}\right)T_{pub}$ holds. If not, the message will be rejected. Otherwise, SVs will accept the broadcast message and store ζ locally for the subsequent derivation of the group identifier.

### 4.3. Authentication

**Figure 5:**
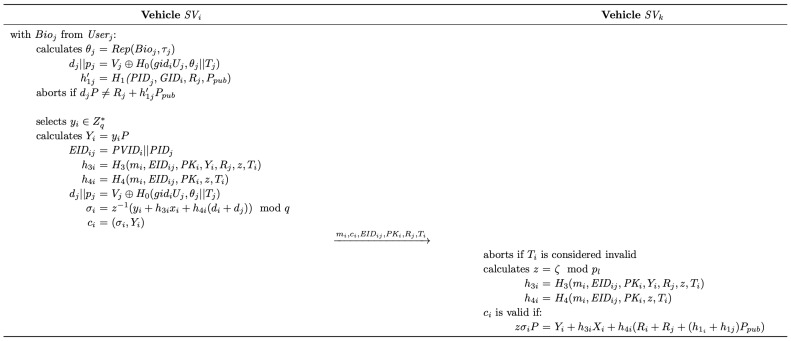
The authentication process of the proposed scheme.

By providing the biometric information, registered users can log into any SV within the same pre-selected target category. Subsequently, the authenticated SVs can establish communications with signature generation and verification. The detailed process of the authentication phase is illustrated in Figure 5.

#### 4.3.1. User Login

Suppose that ${\mathrm{User}}_{j}$ is requesting to log in to ${\mathrm{SV}}_{i}$, where the target identifier $GID_{j}=GID_{i}$.

(1) Upon receiving the biometric data $Bio_{j}$ from ${\mathrm{User}}_{j}$, ${\mathrm{SV}}_{i}$ utilizes the fuzzy extractor to obtain $\theta_{j}=Rep\left(Bio_{j},\tau_{j}\right)$.
(2) Next, ${\mathrm{SV}}_{i}$ calculates $d_{j}\left|\right|p_{j}=V_{j}⊕H_{0}(gid_{i}U_{j},\theta_{j}\left|\right|T_{j})$, $h_{1j}^{\prime }=H_{1}\left({\mathrm{PID}}_{j},GID_{i},R_{j},P_{pub}\right)$ and checks whether the equation $d_{j}P=R_{j}+h_{1j}^{\prime }P_{pub}$ holds. If not, ${\mathrm{SV}}_{i}$ rejects the request. Otherwise, ${\mathrm{User}}_{j}$ succeeds in the login process.

#### 4.3.2. Signing

Prior to generating the signature for message $m_{i}$, ${\mathrm{SV}}_{i}$ extracts the group identifier from the locally stored ζ by calculating $z=\zeta \;\mathrm{mod}\;p_{j}$. Subsequently, ${\mathrm{SV}}_{i}$ proceeds with the signing process, which is detailed as follows:

(1) ${\mathrm{SV}}_{i}$ randomly selects $y_{i}\in Z_{q}^{*}$ and calculates $Y_{i}=y_{i}P$.
(2) ${\mathrm{SV}}_{i}$ then acquires the hash values $h_{3i},h_{4i}$ by calculating $h_{3i}=H_{3}\left(m_{i},EID_{ij},{\mathrm{PK}}_{i},Y_{i},R_{j},z,T_{i}\right)$ and $h_{4i}=H_{4}\left(m_{i},{\mathrm{EID}}_{ij},{\mathrm{PK}}_{i},z,T_{i}\right)$, where $T_{i}$ denotes the current timestamp and ${\mathrm{EID}}_{ij}={\mathrm{PVID}}_{i}\left|\right|{\mathrm{PID}}_{j}$.
(3) ${\mathrm{SV}}_{i}$ calculates $\sigma_{i}=z^{-1}\left(y_{i}+h_{3i}x_{i}+h_{4i}\left(d_{i}+d_{j}\right)\right)\;\mathrm{mod}\;q$, where $z^{-1}$ denotes the multiplicative inverse of *z* on $Z_{q}^{*}$.
(4) Finally, ${\mathrm{SV}}_{i}$ constructs the signature $c_{i}=\left(\sigma_{i},Y_{i}\right)$ and sends the tuple $\left(m_{i},c_{i},{\mathrm{EID}}_{ij},{\mathrm{PK}}_{i},R_{j},T_{i}\right)$ to the receiver.

#### 4.3.3. Verification

Upon receiving the tuple from the sender, ${\mathrm{SV}}_{i}$, a logged-in SV can verify the signature and proceed with authentication.

(1) Upon receiving the message, vehicle ${\mathrm{SV}}_{k}$ will first check its freshness and will abort it if the timestamp is considered invalid. Then, as mentioned in the signing process, ${\mathrm{SV}}_{k}$ will extract *z* from ξ stored locally by calculating $z=\zeta \;\mathrm{mod}\;p_{l}$, where $p_{l}$ denotes the identifier update secret obtained during the user login phase.
(2) With the received parameters, ${\mathrm{SV}}_{k}$ calculates $h_{3i}=H_{3}\left(m_{i},{\mathrm{EID}}_{ij},{\mathrm{PK}}_{i},Y_{i},R_{j},z,T_{i}\right)$ and $h_{4i}=H_{4}\left(m_{i},{\mathrm{EID}}_{ij},{\mathrm{PK}}_{i},z,T_{i}\right)$.
(3) Finally, ${\mathrm{SV}}_{k}$ checks whether the equation $z\sigma_{i}P=Y_{i}+h_{3i}X_{i}+h_{4i}\left(R_{i}+R_{j}+\left(h_{1_{i}}+h_{1j}\right)P_{pub}\right)$ holds. If not, the authentication process fails. Otherwise, ${\mathrm{SV}}_{k}$ accepts $c_{i}$ as a valid signature from ${\mathrm{SV}}_{i}$.

### 4.4. User Logout

When the service of ${\mathrm{User}}_{j}$ terminates, ${\mathrm{SV}}_{i}$ erases the credentials and sends a logout notification containing ${\mathrm{User}}_{j}$’s information to TA and KGC. Upon receiving the logout message, TA and KGC generate the new credential and identifier update secret for ${\mathrm{User}}_{j}$ to enable future vehicle usage.

1. Upon service termination of ${\mathrm{User}}_{j}$, ${\mathrm{SV}}_{i}$ randomly selects $\kappa \in Z_{q}^{*}$ and calculates χ=κP and $\iota =\kappa +H_{2}\left({\mathrm{PID}}_{j},\chi ,X_{i},T_{l}\right)x_{i}$, where $T_{l}$ denotes the current timestamp. Then, ${\mathrm{SV}}_{i}$ transmits $\left({\mathrm{PID}}_{j},\chi ,\iota ,T_{l}\right)$ to TA and KGC. When receiving the message, TA and KGC can verify its authenticity by checking whether the equation $\iota P=\chi +H_{2}\left({\mathrm{PID}}_{j},\chi ,X_{i},T_{l}\right)X_{i}$ holds.
2. After successful verification, based on the received ${\mathrm{PID}}_{j}$, KGC randomly selects $r_{j}^{\prime }\in Z_{q}^{*}$ and generates the corresponding credential $d_{j}^{\prime }$ for ${\mathrm{User}}_{j}$ with operations described in the *User registration* section, which are then transmitted to TA.
3. According to the received information, TA retrieves the relevant parameters from the list *L*, selects a new identifier update secret $p_{j}^{\prime }\in Z_{prime}$, and calculates $V_{j}^{\prime }\;=\;(d_{j}^{\prime }\left|\right|p_{j}^{\prime })\;⊕\;H_{0}(u_{j}{\mathrm{GID}}_{j},\theta_{j}\left|\right|T_{m})$, where $T_{m}$ is the current timestamp.
4. TA then updates the recorded parameters in list *L* with $\rho_{j}^{\prime }$, which is calculated based on the newly generated $p_{j}^{\prime }$.
5. Finally, TA updates $\phi_{g}$ and Ω with $\phi_{g_{new}}=\phi_{g}\times \frac{p_{j}^{\prime }}{p_{j}}$, $\Omega^{\prime }=\Omega +\rho_{j}^{\prime }-\rho_{j}$, and broadcasts $\left({\mathrm{PID}}_{j},R_{j}^{\prime },\tau_{j},V_{j}^{\prime },U_{j},T_{m}\right)$ via a public channel. Additionally, TA performs the operations described in the *Group identifier distribution* section to prevent potential illegal access with the outdated group identifier.

### 4.5. Revocation

Upon the detection of irregular behaviors, TA is responsible for revealing the real identity of the malicious user and revoking the corresponding user from the system by generating a new group identifier. Suppose the user to be revoked is ${\mathrm{User}}_{r}$.

1. TA first acquires the corresponding $\rho_{r}$ by searching the maintained list *L* and updates Ω by calculating $\Omega^{\prime }=\Omega -\rho_{r}$.
2. Next, TA randomly selects $z^{\prime },\psi \in Z_{q}^{*}$ and calculates $\zeta^{\prime }=z^{\prime }\Omega^{\prime }$, Ψ=ψP, and $\nu =\psi +H_{2}\left(\zeta^{\prime },\Psi ,T_{pub},T_{r}\right)t$, where $T_{r}$ denotes the current timestamp.
3. TA then broadcasts $\left(\zeta^{\prime },\Psi ,\nu ,T_{r}\right)$ to all SVs within the system.
4. Upon receiving the update message from TA, each SV first checks the timestamp of the message. If the message is fresh, then vehicles will continue to check whether the equation $\nu P=\Psi +H_{2}\left(\zeta^{\prime },\Psi ,T_{pub},T_{r}\right)T_{pub}$ holds. If not, then the update process is aborted. Otherwise, a new group identifier can be calculated with $z^{\prime }=\zeta^{\prime }\;\mathrm{mod}\;p_{user}$, where $p_{user}$ denotes the individual identifier update secret held by each SV within the system.

## 5. Security Analysis

In this section, we first prove the existential unforgeability of our scheme under the random oracle model. Then, we analyze the security features of our scheme utilizing the defined security model.

### 5.1. Security Proof

**Figure 6:**
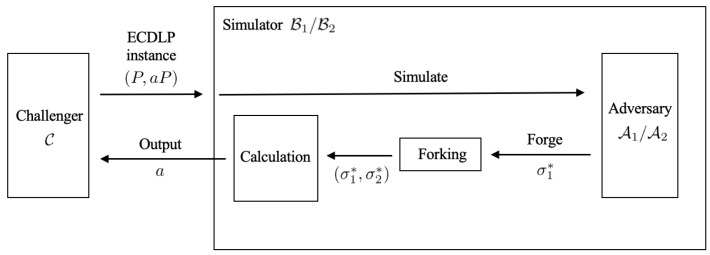
The construction of the security reduction process.

The proofs of Theorem 1 and Theorem 2 are based on a security reduction from the ECDLP, as illustrated in Figure 6. Specifically, we design simulators $B_{1}$ and $B_{2}$ to interact with two distinct types of adversaries, $A_{1}$ and $A_{2}$, respectively. Given an ECDLP instance (P,aP) provided by the challenger C, the simulator B engages with the adversary A in a simulated environment to produce a forged signature. Subsequently, by applying the Forking Lemma [46,47], B is able to obtain a second forgery corresponding to a different random oracle query made by A. From these two distinct forgeries, the simulator can extract and output the solution to the given ECDLP instance, thus completing the reduction.

**Theorem** **1.** *With the hardness assumption of the ECDLP, our proposed ECACS scheme realizes EUF-CMA security against Type-I adversaries under the random oracle model.*

The following Lemma 1 concludes the proof of Theorem 1.

**Lemma** **1.** *If there exists a Type-I adversary $A_{1}$ who can successfully forge a valid signature with obvious advantage $\epsilon_{1}$, then a simulator $B_{1}$ can be constructed to solve the ECDLP with non-negligible advantage $\frac{\left(e-1\right)\epsilon_{1}}{e^{2}\cdot Q_{ur}\left(Q_{h_{4}}+Q_{sig}+1\right)}$, where $Q_{ur}$, $Q_{sig}$, and $Q_{h_{4}}$ denote the times of User Registration Query, Sign Query, and $H_{4}$ Query, respectively.*

**Proof.**  For the proof of Lemma 1, the challenger C will first set up an ECDLP instance $\left\{P,aP|a\in Z_{p}^{*}\right\}$ and send it to $B_{1}$. Then, $B_{1}$ will play the following game with $A_{1}$ in order to acquire the solution to the ECDLP instance. The detailed process is as follows:

Setup: $B_{1}$ sets $P_{pub}=aP$ and sends the system parameters $\left\{G,q,P,P_{pub},H_{0},H_{1},H_{2},H_{3},H_{4},\Omega \right\}$ to $A_{1}$. Meanwhile, $B_{1}$ maintains six lists to record the query results, namely $L_{v},L_{u},L_{1},L_{2},L_{3},L_{4}$.

Query Stage: In this stage, $A_{1}$ will adaptively submit the following queries.

(1) Hash Query: $B_{1}$ will respond to the hash queries from $A_{1}$ as follows:
  (a) $H_{1}$ Query: When receiving an $H_{1}$ hash query with $\left(\mathrm{str},G,R_{i},P_{pub}\right)$, if $\left(\mathrm{str},G,R_{i},P_{pub},h_{1_{i}}\right)\in L_{1}$, $B_{1}$ returns $h_{1i}$. Otherwise, $B_{1}$ selects $h_{1_{i}}\in Z_{q}^{*}$, adds the tuple $\left(\mathrm{str},G,R_{i},P_{pub},h_{1_{i}}\right)$ to $L_{1}$, and then returns $h_{1_{i}}$. Note that the str and G here generally represent a random string and a point on the defined elliptic curve.
  (b) $H_{2}$ Query: When receiving an $H_{2}$ hash query with $\left(\zeta ,W,P_{pub},T_{z}\right)$, if $\left(\zeta ,W,P_{pub},T_{z},h_{2_{i}}\right)\in L_{2}$, $B_{1}$ returns $h_{2_{i}}$. Otherwise, $B_{1}$ selects $h_{2_{i}}\in Z_{q}^{*}$, adds the tuple $\left(\zeta ,W,P_{pub},T_{z},h_{2_{i}}\right)$ to $L_{2}$, and then returns $h_{2_{i}}$.
  (c) $H_{3}$ Query: When receiving an $H_{3}$ hash query with $\left(m_{i},{\mathrm{EID}}_{ij},{\mathrm{PK}}_{i},Y_{i},R_{j},z,T_{i}\right)$, if the corresponding tuple $\left(m_{i},{\mathrm{EID}}_{ij},{\mathrm{PK}}_{i},Y_{i},R_{j},z,T_{i},h_{3_{i}}\right)\in L_{3}$, then $B_{1}$ returns $h_{3_{i}}$. Otherwise, $B_{1}$ selects $h_{3_{i}}\in Z_{q}^{*}$, adds $\left(m_{i},{\mathrm{EID}}_{ij},{\mathrm{PK}}_{i},Y_{i},R_{j},z,T_{i},h_{3_{i}}\right)$ to $L_{3}$, and then returns $h_{3_{i}}$.
  (d) $H_{4}$ Query: When receiving an $H_{4}$ hash query with $\left(m_{i},{\mathrm{EID}}_{ij},{\mathrm{PK}}_{i},z,T_{i}\right)$, if the corresponding tuple $\left(m_{i},{\mathrm{EID}}_{ij},{\mathrm{PK}}_{i},z,T_{i},h_{4_{i}}\right)\in L_{4}$, then $B_{1}$ returns $h_{4_{i}}$. Otherwise, $B_{1}$ selects $h_{4_{i}}\in Z_{q}^{*}$, adds $\left(m_{i},{\mathrm{EID}}_{ij},{\mathrm{PK}}_{i},z,T_{i},h_{4_{i}}\right)$ to $L_{4}$, and then returns $h_{4_{i}}$.
(2) Vehicle Registration Query: When $B_{1}$ receives a Vehicle Registration Query from $A_{1}$ with ${\mathrm{PVID}}_{i}$ as input, if ${\mathrm{PVID}}_{i}$ exists in $L_{v}$, $B_{1}$ returns ⊥. Otherwise, $B_{1}$ selects $x_{i},d_{i},gid_{i},h_{1_{i}}\in Z_{q}^{*}$ and calculates $R_{i}=d_{i}P-h_{1_{i}}P_{pub}$, $X_{i}=x_{i}P$ and $GID_{i}=gid_{i}P$. Then, $B_{1}$ adds $\left({\mathrm{PVID}}_{i},x_{i},X_{i},d_{i},R_{i},gid_{i},GID_{i}\right)$ to $L_{v}$ and $\left({\mathrm{PVID}}_{i},X_{i},R_{i},P_{pub},h_{1_{i}}\right)$ to $L_{1}$.
(3) User Registration Query: When $B_{1}$ receives a User Registration Query from $A_{1}$ with ${\mathrm{PID}}_{j}$ as input, if ${\mathrm{PID}}_{j}$ exists in $L_{u}$, $B_{1}$ returns ⊥. Otherwise, $B_{1}$ continues the game as follows:
  - If ${\mathrm{PID}}_{j}={\mathrm{PID}}^{*}$, $B_{1}$ selects $r_{j},h_{1_{i}}\in Z_{q}^{*}$ and calculates $R_{j}=r_{j}P$. Then, $B_{1}$ adds the tuple $\left({\mathrm{PID}}_{j},GID_{j},⊥,R_{j}\right)$ to $L_{u}$ and the tuple $\left({\mathrm{PID}}_{j},GID_{j},R_{j},P_{pub},h_{1_{j}}\right)$ to $L_{1}$, where $GID_{j}$ is selected from the existing records in $L_{v}$.
  - If $\mathrm{PID}j\neq {\mathrm{PID}}^{*}$, $B_{1}$ selects $d_{j},h_{1_{i}}\in Z_{q}^{*}$ and calculates $R_{j}=d_{j}P-h_{1_{i}}P_{pub}$. Then, $B_{1}$ adds $\left({\mathrm{PID}}_{j},GID_{j},d_{j},R_{j}\right)$ to $L_{u}$ and $\left({\mathrm{PID}}_{j},GID_{j},R_{j},P_{pub},h_{1_{j}}\right)$ to $L_{1}$, where $GID_{j}$ is selected from the existing records in $L_{v}$.
(4) Reveal Vehicle Partial Key Query: When $B_{1}$ receives a Reveal Vehicle Partial Key Query from $A_{1}$ with ${\mathrm{PVID}}_{i}$ as input, if the tuple $\left(PVID_{i},x_{i},X_{i},d_{i},R_{i},gid_{i},GID_{i}\right)\in L_{v}$, $B_{1}$ returns the corresponding $d_{i}$. Otherwise, $B_{1}$ first executes the Vehicle Registration Query with ${\mathrm{PVID}}_{i}$ as input and then returns the corresponding $d_{i}$.
(5) Reveal User Private Key Query: When $B_{1}$ receives a Reveal User Private Key Query from $A_{1}$ with ${\mathrm{PID}}_{j}$ as input, if ${\mathrm{PID}}_{j}={\mathrm{PID}}^{*}$, $B_{1}$ aborts the game. Otherwise, $B_{1}$ continues the game as follows:
  - If ${\mathrm{PID}}_{j}\in L_{u}$, $B_{1}$ returns the corresponding $d_{j}$.
  - If ${\mathrm{PID}}_{j}\notin L_{u}$, $B_{1}$ first executes the User Registration Query and then returns the corresponding $d_{j}$.
(6) Reveal Vehicle Secret Query: When $B_{1}$ receives a Reveal Vehicle Secret Query from $A_{1}$ with $PVID_{i}$ as input, $B_{1}$ returns $x_{i}$ if $\left(PVID_{i},x_{i},X_{i},d_{i},R_{i},gid_{i},GID_{i}\right)\in L_{v}$. Otherwise, $B_{1}$ first executes the Vehicle Registration Query with $PVID_{i}$ as input and then returns the corresponding $x_{i}$.
(7) Reveal Vehicle Public Key Query: When $B_{1}$ receives a Reveal Public Key Query from $A_{1}$ with ${\mathrm{PVID}}_{i}$ as input, if $\left(PVID_{i},x_{i},X_{i},d_{i},R_{i},gid_{i},GID_{i}\right)\in L_{v}$, $B_{1}$ returns the corresponding $\left(X_{i},R_{i}\right)$. Otherwise, $B_{1}$ first executes the Vehicle Registration Query with ${\mathrm{PVID}}_{i}$ as input and then returns the corresponding $\left(X_{i},R_{i}\right)$.
(8) Reveal User Public Key Query: When $B_{1}$ receives a Reveal User Public Key Query from $A_{1}$ with ${\mathrm{PID}}_{j}$ as input, if $\left({\mathrm{PID}}_{j},GID_{j},d_{j},R_{j}\right)\in L_{u}$, $B_{1}$ returns the corresponding $R_{j}$. Otherwise, $B_{1}$ first executes the User Registration Query with ${\mathrm{PID}}_{j}$ as input and then returns the corresponding $R_{j}$.
(9) Replace Vehicle Public Key Query: When $B_{1}$ receives a Replace Vehicle Public Key Query from $A_{1}$ with $\left(PVID_{i},X_{i}^{\prime },R_{i}^{\prime }\right)$ as input, $B_{1}$ sets $\left(X_{i}^{\prime },R_{i}^{\prime }\right)$ as ${\mathrm{PVID}}_{i}$s new public key and updates the record in $L_{v}$.
(10) Replace User Public Key Query: When $B_{1}$ receives a Replace User Public Key Query from $A_{1}$ with $\left({\mathrm{PID}}_{j},R_{j}^{\prime }\right)$ as input, $B_{1}$ sets $R_{i}^{\prime }$ as ${\mathrm{PID}}_{j}$’s new public key and updates the record in $L_{u}$.
(11) Sign Query: When $B_{1}$ receives a Sign Query from $A_{1}$ with $\left({\mathrm{PVID}}_{i},{\mathrm{PID}}_{j},m_{i}\right)$ as input, $B_{1}$ first checks whether ${\mathrm{PVID}}_{i}$ and ${\mathrm{PID}}_{j}$ exist in $L_{v}$ and $L_{u}$. If not, $B_{1}$ first executes the Vehicle Registration Query and User Registration Query with the input identities. Then, after acquiring the corresponding records, $B_{1}$ continues the game as follows:
  - If ${\mathrm{PID}}_{j}\neq {\mathrm{PID}}^{*}$, $B_{1}$ selects $z,y_{i},h_{3_{i}},h_{4_{i}}\in Z_{q}^{*}$ and obtains $h_{1_{i}}$, $h_{1_{j}}$ from $L_{1}$. Then, $B_{1}$ calculates $Y_{i}=y_{i}P$, $\sigma_{i}=z^{-1}\left(y_{i}+h_{3_{i}}x_{i}+h_{4_{i}}\left(d_{i}+d_{j}\right)\right)$. Then, $B_{1}$ adds $\left(m_{i},{\mathrm{EID}}_{ij},GID_{j},{\mathrm{PK}}_{i},Y_{i},R_{j},z,T_{z},h_{3_{i}}\right)$ to $L_{3}$ and $\left(m_{i},{\mathrm{EID}}_{ij},GID_{j},{\mathrm{PK}}_{i},z,T_{z},h_{4_{i}}\right)$ to $L_{4}$, where ${\mathrm{EID}}_{ij}=PVID_{i}\left|\right|{\mathrm{PID}}_{j}$. Finally, $B_{1}$ returns $\left(\sigma_{i},Y_{i},T_{z}\right)$ to $A_{1}$.
  - If ${\mathrm{PID}}_{j}={\mathrm{PID}}^{*}$, $B_{1}$ selects $z,y_{i},h_{3_{i}},h_{4_{i}}\in Z_{q}^{*}$ and obtains $h_{1_{i}}$, $h_{1_{j}}$ from $L_{1}$. Then, $B_{1}$ sets $\sigma_{i}=z^{-1}y_{i}$, $Y_{i}=z\sigma_{i}P-h_{3_{i}}X_{i}-h_{4_{i}}\left(R_{i}+R_{j}+h_{1_{ij}}P_{pub}\right)$, where $h_{1_{ij}}=\left(h_{1_{i}}+h_{1_{j}}\right)$. Then, $B_{1}$ adds $\left(m_{i},{\mathrm{EID}}_{ij},GID_{j},{\mathrm{PK}}_{i},Y_{i},R_{j},z,T_{z},h_{3_{i}}\right)$ to $L_{3}$ and $\left(m_{i},{\mathrm{EID}}_{ij},GID_{j},{\mathrm{PK}}_{i},z,T_{z},h_{4_{i}}\right)$ to $L_{4}$, where ${\mathrm{EID}}_{ij}={\mathrm{PVID}}_{i}\left|\right|{\mathrm{PID}}_{j}$. Finally, $B_{1}$ returns $\left(\sigma_{i},Y_{i},T_{z}\right)$ to $A_{1}$.

Forgery Stage: After all the queries have been made and replied to, $A_{1}$ outputs a forged signature $\left(\sigma_{1}^{*},Y_{i}\right)$ for the identity-message pair $\left({\mathrm{PVID}}_{i},{\mathrm{PID}}_{j}^{*},m^{*}\right)$. If ${\mathrm{PID}}_{j}^{*}\neq {\mathrm{PID}}^{*}$, $B_{1}$ outputs ⊥ and aborts the game. Otherwise, according to the Forking Lemma, $B_{1}$ can rewind the game process and make $A_{1}$ output another forged signature $\left(\sigma_{2}^{*},Y_{i}\right)$ by changing the output of $H_{4}$. Hence, we have

$$\left\{\begin{array}{c} \sigma_{1}^{*}=y_{i}^{*}+h_{3}^{*}x_{i}^{*}+h_{4}^{*}\left(d_{i}^{*}+r_{j}^{*}+h_{1_{ij}}^{*}a\right)\\ \sigma_{2}^{*}=y_{i}^{*}+h_{3}^{*}x_{i}^{*}+h_{4}^{{*}^{\prime }}\left(d_{i}^{*}+r_{j}^{*}+h_{1_{ij}}^{*}a\right) \end{array}\right.$$

From the above equations, $B_{1}$ outputs $a=\frac{1}{h_{1_{ij}}^{*}}\left(\frac{\sigma_{1}^{*}-\sigma_{2}^{*}}{h_{4}^{*}-h_{4}^{{*}^{\prime }}}-d_{i}^{*}-r_{j}^{*}\right)$ as the solution to the ECDLP instance.

Let $E_{1}$ denote the event that the game does not abort in the Query Stage, $E_{2}$ denote the event that the game does not abort in the Forge Stage, and $E_{3}$ denote the event that $A_{1}$ successfully outputs two valid forged signatures in the game. Then the advantage of $B_{1}$ solving the ECDLP instance can be presented with $Pr\left[\epsilon_{B_{1}}\right]=Pr\left[E_{1}\wedge E_{2}\wedge E_{3}\right]=Pr\left[E_{1}\right]Pr\left[E_{2}|E_{1}\right]Pr\left[E_{3}|E_{1}\wedge E_{2}\right]$. Furthermore, we have $Pr\left[E_{1}\right]\geq {\left(1-\frac{1}{Q_{ur}}\right)}^{\left(Q_{upk}+Q_{sig}\right)}$, $Pr\left[E_{2}|E_{1}\right]\geq \frac{1}{Q_{ur}}$, where $Q_{ur}$ denotes the times of User Registration Query, $Q_{upk}$ denotes the times of Reveal User Private Key Query, and $Q_{sig}$ denotes the times of Sign Query.

According to the Forking Lemma, we have $Pr\left[E_{3}|E_{1}\wedge E_{2}\right]\geq \left(1-\frac{1}{e}\right)\frac{\epsilon_{1}}{Q_{h_{4}}+Q_{sig}+1}$, where $\epsilon_{1}$ represents the advantage that $A_{1}$ can successfully forge a signature, and $Q_{h_{4}}$ represents the times of $H_{4}$ Query.

Therefore, $B_{1}$’s advantage of solving the ECDLP instance is

$$\begin{array}{cc} Pr[\epsilon_{B_{1}}] & =Pr[E_{1}\wedge E_{2}\wedge E_{3}]\\ \; & =Pr\left[E_{1}\right]Pr\left[E_{2}|E_{1}\right]Pr\left[E_{3}|E_{1}\wedge E_{2}\right]\\ \; & \geq \left[{\left(1-\frac{1}{Q_{ur}}\right)}^{\left(Q_{upk}+Q_{sig}\right)}\right]\frac{1}{Q_{ur}}\left(1-\frac{1}{e}\right)\frac{\epsilon_{1}}{Q_{h_{4}}+Q_{sig}+1}\\ \; & \geq \frac{\left(e-1\right)\epsilon_{1}}{e^{2}\cdot Q_{ur}\left(Q_{h_{4}}+Q_{sig}+1\right)} \end{array}$$

Thus, if there exists an adversary $A_{1}$ who can successfully forge a signature with advantage $\epsilon_{1}$, then $B_{1}$ can output the solution to the ECDLP instance with probability $\frac{\left(e-1\right)\epsilon_{1}}{e^{2}\cdot Q_{ur}\left(Q_{h_{4}}+Q_{sig}+1\right)}$. □

**Theorem** **2.** *With the hardness assumption of the ECDLP, our proposed ECACS scheme realizes EUF-CMA security against Type-II adversaries under the random oracle model.*

The following Lemma 2 concludes the proof of Theorem 2.

**Lemma** **2.** *If there exists a Type-II adversary $A_{2}$ who can successfully forge a valid signature with obvious advantage $\epsilon_{2}$, then a simulator $B_{2}$ can be constructed to solve the ECDLP with non-negligible advantage $\left(1-\frac{1}{e}\right)\frac{\epsilon_{2}}{e\cdot Q_{vr}Q_{h_{3}}}$, where $Q_{vr}$ and $Q_{h_{3}}$ denote the times of Vehicle Registration Query and $H_{3}$ Query, respectively.*

**Proof.**  For the proof of Lemma 2, the challenger C will set up an ECDLP instance $\left\{P,bP|b\in Z_{p}^{*}\right\}$ and send it to $B_{2}$. Then, $B_{2}$ will play the following game with $A_{2}$ in order to acquire the solution to the ECDLP instance. The detailed process is as follows:

Setup: $B_{2}$ sets $P_{pub}=sP$ and sends *s* and the parameters $\left\{G,q,P,P_{pub},H_{0},H_{1},H_{2},H_{3},H_{4},\Omega \right\}$ to $A_{2}$. Meanwhile, $B_{2}$ maintains six lists to record the query results, namely $L_{v},L_{u},L_{1},L_{2},L_{3},L_{4}$.

Query Stage: In this stage, $A_{2}$ will adaptively submit the following queries.

(1) Hash Query: $B_{2}$ answers the Hash Query the same as in Game I.
(2) Vehicle Registration Query: When $B_{2}$ receives a Vehicle Registration Query from $A_{2}$ with ${\mathrm{PVID}}_{i}$ as input, if ${\mathrm{PVID}}_{i}$ exists in $L_{v}$, $B_{1}$ returns ⊥. Otherwise, continue the game as follows.
  - If ${\mathrm{PVID}}_{i}={\mathrm{PVID}}^{*}$, $B_{2}$ selects $r_{i},gid_{i},h_{1_{i}}\in Z_{q}^{*}$ and calculates $R_{i}=r_{i}P$, $GID_{i}=gid_{i}P$ and $d_{i}=r_{i}+h_{1_{i}}s$. Then, $B_{2}$ sets $X_{i}=bP$ and adds the tuple $\left({\mathrm{PVID}}_{i},⊥,X_{i},d_{i},R_{i},gid_{i},GID_{i}\right)$ to $L_{v}$ and $\left({\mathrm{PVID}}_{i},X_{i},R_{i},P_{pub},h_{1_{i}}\right)$ to $L_{1}$.
  - If ${\mathrm{PVID}}_{i}\neq {\mathrm{PVID}}^{*}$, $B_{2}$ selects $x_{i},r_{i},gid_{i},h_{1_{i}}\in Z_{q}^{*}$ and calculates $R_{i}=r_{i}P$, $X_{i}=x_{i}P$, $GID_{i}=gid_{i}P$ and $d_{i}=r_{i}+h_{1_{i}}s$. Then, $B_{1}$ adds the tuple $\left({\mathrm{PVID}}_{i},x_{i},X_{i},d_{i},R_{i},gid_{i},GID_{i}\right)$ to $L_{v}$ and $\left({\mathrm{PVID}}_{i},X_{i},R_{i},P_{pub},h_{1_{i}}\right)$ to $L_{1}$.
(3) User Registration Query: When $B_{1}$ receives a User Registration Query from $A_{1}$ with ${\mathrm{PID}}_{j}$ as input, if ${\mathrm{PID}}_{j}$ exists in $L_{u}$, $B_{1}$ returns ⊥. Otherwise, $B_{2}$ selects $d_{j},h_{1_{i}}\in Z_{q}^{*}$ and calculates $R_{j}=d_{j}P-h_{1_{i}}P_{pub}$. Then, $B_{2}$ adds the tuple $\left({\mathrm{PID}}_{j},GID_{j},d_{j},R_{j}\right)$ to $L_{u}$ and the tuple $\left({\mathrm{PID}}_{j},GID_{j},R_{j},P_{pub},h_{1_{j}}\right)$ to $L_{1}$, where $GID_{j}$ is selected from the existing records in $L_{v}$.
(4) Reveal User Private Key Query: When $B_{2}$ receives a Reveal User Private Key Query from $A_{2}$ with ${\mathrm{PID}}_{j}$ as input, if the tuple $\left({\mathrm{PID}}_{j},GID_{j},d_{j},R_{j}\right)\in L_{u}$, B2 returns the corresponding $d_{j}$. Otherwise, $B_{2}$ first executes the User Registration Query with ${\mathrm{PID}}_{j}$ as input and then returns the corresponding $d_{j}$.
(5) Reveal Vehicle Secret Query: When $B_{2}$ receives a Reveal Vehicle Secret Query from $A_{2}$ with ${\mathrm{PVID}}_{i}$ as input, if ${\mathrm{PVID}}_{i}={\mathrm{PVID}}^{*}$, $B_{2}$ aborts the game. Otherwise, $B_{2}$ continues the game as follows:
  - If ${\mathrm{PVID}}_{i}\in L_{v}$, $B_{2}$ returns the corresponding $x_{i}$.
  - If ${\mathrm{PVID}}_{i}\notin L_{v}$, $B_{2}$ first executes the Vehicle Registration Query and then returns the corresponding $x_{i}$.
(6) Reveal Vehicle Public Key Query: When $B_{2}$ receives a Reveal Public Key Query from $A_{2}$ with ${\mathrm{PVID}}_{i}$ as input, if $\left({\mathrm{PVID}}_{i},x_{i},X_{i},d_{i},R_{i},gid_{i},GID_{i}\right)\in L_{v}$, $B_{2}$ returns the corresponding $\left(X_{i},R_{i}\right)$. Otherwise, $B_{2}$ first executes the Vehicle Registration Query with ${\mathrm{PVID}}_{i}$ as input and then returns the corresponding $\left(X_{i},R_{i}\right)$.
(7) Reveal User Public Key Query: When $B_{2}$ receives a Reveal User Public Key Query from $A_{2}$ with ${\mathrm{PID}}_{j}$ as input, if $\left({\mathrm{PID}}_{j},GID_{j},d_{j},R_{j}\right)\in L_{u}$, $B_{2}$ returns the corresponding $R_{j}$. Otherwise, $B_{2}$ first executes the User Registration Query with ${\mathrm{PID}}_{j}$ as input and then returns the corresponding $R_{j}$.
(8) Sign Query: When $B_{2}$ receives a Sign Query from $A_{2}$ with $\left({\mathrm{PVID}}_{i},{\mathrm{PID}}_{j},m_{i}\right)$ as input, $B_{2}$ first checks whether ${\mathrm{PVID}}_{i}$ and ${\mathrm{PID}}_{j}$ exist in $L_{v}$ and $L_{u}$. If not, $B_{2}$ first executes the Vehicle Registration Query and User Registration Query with the input identities. Then, after acquiring the corresponding records, $B_{2}$ continues the game as follows:
  - If ${\mathrm{PVID}}_{j}\neq {\mathrm{PVID}}^{*}$, $B_{2}$ selects $z,y_{i},h_{3_{i}},h_{4_{i}}\in Z_{q}^{*}$ and obtains $h_{1_{i}}$, $h_{1_{j}}$ from $L_{1}$. Then, $B_{2}$ calculates $Y_{i}=y_{i}P$, $\sigma_{i}=z^{-1}\left(y_{i}+h_{3_{i}}x_{i}+h_{4_{i}}\left(d_{i}+d_{j}\right)\right)$. Then, $B_{2}$ adds $\left(m_{i},{\mathrm{EID}}_{ij},GID_{j},{\mathrm{PK}}_{i},Y_{i},R_{j},z,T_{z},h_{3_{i}}\right)$ to $L_{3}$ and $\left(m_{i},{\mathrm{EID}}_{ij},GID_{j},{\mathrm{PK}}_{i},z,T_{z},h_{4_{i}}\right)$ to $L_{4}$. Finally, $B_{2}$ returns $\left(\sigma_{i},Y_{i},T_{z}\right)$ to $A_{2}$.
  - If ${\mathrm{PVID}}_{j}={\mathrm{PVID}}^{*}$, $B_{2}$ selects $z,y_{i},h_{3_{i}},h_{4_{i}}\in Z_{q}^{*}$ and obtains $h_{1_{i}}$, $h_{1_{j}}$ from $L_{1}$. Then, $B_{2}$ sets $\sigma_{i}=z^{-1}y_{i}$ and calculates $Y_{i}=z\sigma_{i}P-h_{3_{i}}X_{i}-h_{4_{i}}\left(R_{i}+R_{j}+h_{1_{ij}}P_{pub}\right)$, where $h_{1_{ij}}=\left(h_{1_{i}}+h_{1_{j}}\right)$. $B_{2}$ then adds $\left(m_{i},{\mathrm{EID}}_{ij},GID_{j},{\mathrm{PK}}_{i},Y_{i},R_{j},z,T_{z},h_{3_{i}}\right)$ to $L_{3}$ and $\left(m_{i},{\mathrm{EID}}_{ij},GID_{j},{\mathrm{PK}}_{i},z,T_{z},h_{4_{i}}\right)$ to $L_{4}$, where ${\mathrm{EID}}_{ij}={\mathrm{PVID}}_{i}\left|\right|{\mathrm{PID}}_{j}$. Finally, $B_{2}$ returns $\left(\sigma_{i},Y_{i},T_{z}\right)$ to $A_{2}$.

Forgery Stage: After all the queries have been made and replied to, $A_{2}$ outputs a forged signature $\left(\sigma_{1}^{*},Y_{i}\right)$ for the identity-message pair $\left({\mathrm{PVID}}_{i},{\mathrm{PID}}_{j}^{*},m^{*}\right)$. If ${\mathrm{PVID}}_{i}^{*}\neq {\mathrm{PVID}}^{*}$, $B_{2}$ outputs ⊥ and aborts the game. Otherwise, according to the Forking Lemma, $B_{2}$ can rewind the game process and make $A_{2}$ output another forged signature $\left(\sigma_{2}^{*},Y_{i}\right)$ by changing the output of $H_{3}$. Hence, we have

$$\left\{\begin{array}{c} \sigma_{1}^{*}=y_{i}^{*}+h_{3}^{{*}^{\prime }}b+h_{4}^{*}\left(d_{i}^{*}+d_{j}^{*}\right)\\ \sigma_{2}^{*}=y_{i}^{*}+h_{3}^{*}b+h_{4}^{*}\left(d_{i}^{*}+d_{j}^{*}\right) \end{array}\right.$$

From the above equations, $B_{1}$ outputs $b=\frac{\sigma_{1}^{*}-\sigma_{2}^{*}}{h_{3}^{*}-h_{3}^{{*}^{\prime }}}$ as the solution to the ECDLP instance.

Let $E_{1}$ denote the event that the game does not abort in Query Stage, $E_{2}$ denote the event that the game does not abort in Forge Stage, and $E_{3}$ denote the event that $A_{2}$ successfully outputs two valid forged signatures in the game. Then the advantage of $B_{2}$ solving the ECDLP instance can be presented with $Pr\left[\epsilon_{B_{1}}\right]=Pr\left[E_{1}\wedge E_{2}\wedge E_{3}\right]=Pr\left[E_{1}\right]Pr\left[E_{2}|E_{1}\right]Pr\left[E_{3}|E_{1}\wedge E_{2}\right]$. Furthermore, we have $Pr\left[E_{1}\right]\geq {\left(1-\frac{1}{Q_{vr}}\right)}^{\left(Q_{rvs}+Q_{sig}+1\right)}$, $Pr\left[E_{2}|E_{1}\right]\geq \frac{1}{Q_{vr}}$, where $Q_{vr}$ denotes the times of Vehicle Registration Query, $Q_{rvs}$ denotes the times of Reveal Vehicle Secret Query, and $Q_{sig}$ denotes the times of Sign Query.

According to the Forking Lemma, we have $Pr\left[E_{3}|E_{1}\wedge E_{2}\right]\geq \left(1-\frac{1}{e}\right)\frac{\epsilon_{2}}{Q_{h_{3}}+Q_{sig}+1}$, where $\epsilon_{2}$ represents the advantage that $A_{2}$ can successfully forge a signature and $Q_{h_{3}}$ represents the times of $H_{3}$ Query.

Therefore, $B_{2}$’s advantage of solving the ECDLP instance is

$$\begin{array}{cc} Pr[\epsilon_{B_{2}}] & =Pr[E_{1}\wedge E_{2}\wedge E_{3}]\\ \; & =Pr\left[E_{1}\right]Pr\left[E_{2}|E_{1}\right]Pr\left[E_{3}|E_{1}\wedge E_{2}\right]\\ \; & \geq \left[{\left(1-\frac{1}{Q_{vr}}\right)}^{(Q_{rvs}+Q_{sig}+1}\right)]\frac{1}{Q_{vr}}\left(1-\frac{1}{e}\right)\frac{\epsilon_{2}}{Q_{h_{3}}+Q_{sig}+1}\\ \; & \geq \frac{\left(e-1\right)\epsilon_{2}}{e^{2}\cdot Q_{vr}\left(Q_{h_{3}}+Q_{sig}+1)\right)} \end{array}$$

Thus, if there exists an adversary $A_{2}$ who can successfully forge a signature with advantage $\epsilon_{2}$, then $B_{2}$ can output the solution to the ECDLP instance with probability $\frac{\left(e-1\right)\epsilon_{2}}{e^{2}\cdot Q_{vr}\left(Q_{h_{3}}+Q_{sig}+1)\right)}$. □

### 5.2. Security Features

In particular, our CLS scheme satisfies the following security objectives: message authenticity and integrity, anonymity, traceability, revocability, and resistance against replay and MITM attacks.

#### 5.2.1. Message Authenticity and Integrity

Based on the existential unforgeability proof presented above, it is computationally infeasible for either Type-I or Type-II adversaries to forge a valid signature. Furthermore, since the corresponding identifier update secrets of revoked users are no longer included in Ω, no revoked user can generate a valid signature with any SV due to the inability to extract the latest group identifier *z*. Consequently, our proposed scheme ensures both the authenticity and integrity of the transmitted message.

#### 5.2.2. Anonymity

In our proposed scheme, the identities utilized by vehicles and users in the authentication process are pseudonyms generated based on their real identities and TA’s secret key *t*. Consequently, no adversary can obtain the real identity of any entity without access to TA’s secret key. Hence, our proposed scheme achieves anonymity.

#### 5.2.3. Traceability

When emergencies occur, TA can reveal the real identity of any entity by calculating ${\mathrm{VID}}_{i}={\mathrm{PVID}}_{i}⊕H_{0}\left(tR_{j},T_{i}\right)$ or ${\mathrm{UID}}_{i}={\mathrm{PID}}_{i}⊕H_{0}(\upsilon T_{pub},\theta_{j}\left|\right|T_{j})$. Thus, our proposed scheme meets the requirement of traceability.

#### 5.2.4. Revocability

In the event of a user with unusual behaviors, it is imperative for TA to promptly revoke the user’s ability to process authentication within the system. By updating the group identifier *z* and distributing it through the application of the Chinese Remainder Theorem, only vehicles authenticated with the current legitimate users can generate valid signatures and perform the verification process. Thus, our proposed scheme effectively supports the revocation feature.

#### 5.2.5. Resistance Against Replay Attacks

In our scheme, the messages transmitted during the signing and verification processes all contain timestamps. Any message that fails to meet the freshness requirements will be rejected. Therefore, our scheme is capable of resisting replay attacks.

#### 5.2.6. Resistance Against MITM Attacks

To launch a man-in-the-middle (MITM) attack, an adversary would first need to forge a valid signature; otherwise, it would not be able to modify the signature it captures. Moreover, based on the existential unforgeability proof provided above, neither Type-I nor Type-II adversaries can forge a valid signature. Therefore, our proposed scheme is resistant to MITM attacks.

## 6. Performance Evaluation

In this section, we provide the performance comparison between our proposed scheme and the related schemes [48,49,50,51,52,53,54]. Given that practical communications among entities are established over wireless channels, necessitating timely and lightweight information transmission, our evaluation primarily focuses on the calculation time cost and information payload size. Specifically, our evaluation encompasses four key aspects: functional properties, computational overhead, communication overhead, and the revocation feature.

### 6.1. Experiment Environment

Our experiments are conducted on a Lenovo personal computer (Lenovo Group Ltd., Beijing, China) equipped with an Intel Core i5-9500T 2.20 GHz CPU and 8 GB of RAM. The standard open-source MIRACL Crypto SDK is employed on the Ubuntu 22.04 operating system to implement the cryptographic operations, with the security parameter λ set to 128 bits to meet the required security standards. The bilinear pairing operations for pairing-based schemes are performed over a Tate pairing-based curve with an embedding degree of 2, while the scalar operations for ECC-based schemes are executed over the secp256r1 curve. The measured results of the operation time cost are presented in Table 3.

### 6.2. Functional Properties

The comparison results of the functionalities of our proposed scheme with other state-of-the-art relatives are illustrated in Table 4. While the scheme proposed in [48] achieves unforgeability, its reliance on bilinear pairings incurs substantial computational overhead. Regarding ECC-based schemes, though the schemes proposed in [49,50] support revocation, they overlook the need for a decoupled user authentication phase. The scheme proposed in [54] utilizes passwords and biometric data to realize the authentication processes for users, but it is based on individual relationships between users and vehicles, which lacks the flexibility required in rental scenarios. In contrast, our scheme utilizes the Chinese Remainder Theorem to achieve user revocation. Furthermore, the fuzzy extractor and category identifier mechanism allow our scheme to support both user decoupling and category-range selective authentication. Hence, our scheme demonstrates superior performance under the smart car-sharing situations.

### 6.3. Computational Overhead

**Figure 7:**
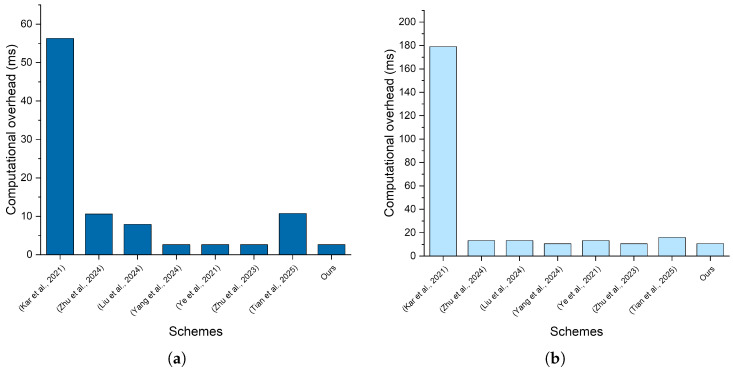
Comparison of the computational overhead of our proposed scheme with related works [48,49,50,51,52,53,54]: (**a**) signature generation phase; (**b**) signature verification phase.

To conduct a comprehensive evaluation, we assess the computational overhead of the schemes by analyzing the time cost associated with signature generation and verification, with the results detailed in Table 5 and Figure 7.

For our scheme, the signature generation process involves one scalar multiplication operation and two general secure hash operations, and the computational cost is $T_{em}+2T_{h}=2.634$ ms. As for signature verification, our proposed scheme requires four ECC scalar multiplication operations, four ECC point addition operations, and four general secure hash operations, which cost $4T_{em}+4T_{ea}+4T_{h}=10.604$ ms. Based on the evaluation, our scheme demonstrates competitive computational efficiency compared with other related schemes. Moreover, our scheme offers additional functionalities, including category-range selection when performing authentication between vehicles and users, as well as the feature of revocation. Therefore, it is reasonable to conclude that our scheme demonstrates outstanding performance in terms of computational overhead.

### 6.4. Communication Overhead

**Figure 8:**
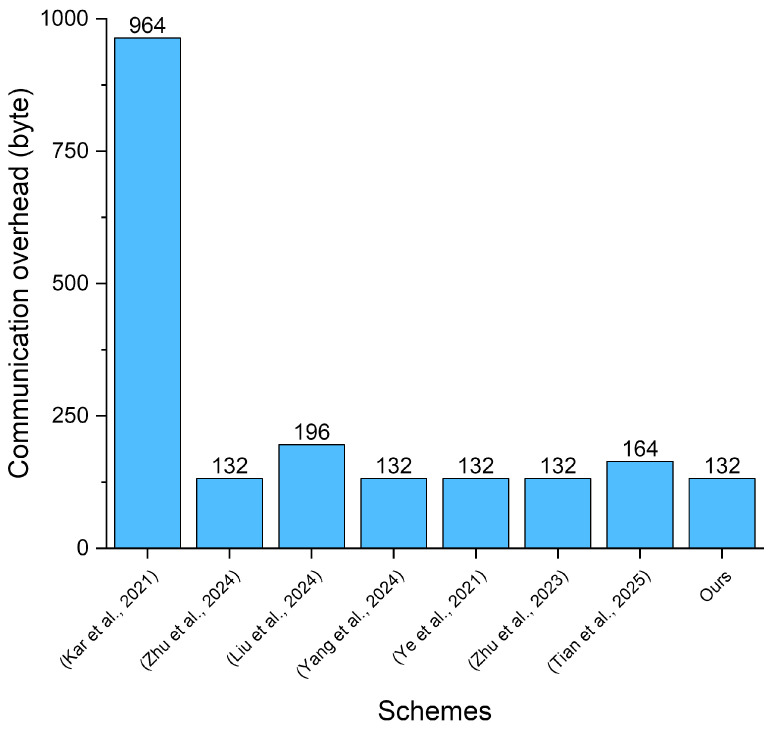
Comparison of the communication overhead of our proposed scheme with related works [48,49,50,51,52,53,54].

In our evaluation, we measure the communication overhead of the related schemes based on the length of the transmitted signature tuples. To meet the requirements of the security parameter, we set the size of elements in $G_{1}$ to 384 bytes, the size of elements in G to 64 bytes, and the size of elements in $Z_{q}^{*}$ to 32 bytes. Considering schemes implying timestamps to resist replay attacks, we set the size of the timestamps to 4 bytes. The comparison results are presented in Table 5 and Figure 8.

In our proposed scheme, the communication overhead for a signature is $\left|G|+2\right|Z_{q}^{*}\left|+|T\right|=132$ bytes. As demonstrated in Table 5 and Figure 4, our scheme is superior to the schemes in [48,54] and is at the same low overhead level as the schemes in [49,51,52,53]. Moreover, while maintaining communication efficiency, our proposed scheme realizes enhanced functionality that increases its practicality in real-world scenarios. Therefore, our scheme outperforms the related scheme in the field of communication overhead.

### 6.5. Revocation Feature

Table 6 presents a comparative analysis of the revocation feature of our proposed scheme against related schemes. Specifically, the schemes in [48,51,53,54] lack the functionality of revocation. Meanwhile, the schemes in [8,52] implement revocation through the use of revocation lists. However, this approach inherently incurs a linear increase in overhead as the system scales and the number of entities grows. Likewise, the revocation mechanism in [50] requires synchronization with the blockchain database, introducing additional latency while still exhibiting linear growth in resource consumption.

In contrast, our proposed scheme leverages the CRT and achieves revocation by broadcasting the group identifier through public channels, thereby ensuring that revocation efficiency remains independent of system scale. This inherent scalability makes our scheme particularly suitable for large-scale applications.

## 7. Conclusions

In this paper, we introduced an enhanced certificateless authentication scheme tailored for smart car-sharing environments. By incorporating biometric information and leveraging the Chinese Remainder Theorem for user membership management, our scheme provides a lightweight, decoupled, and fine-grained authentication mechanism between users and vehicles, eliminating the need for user-side computation. The implementation of category identifiers further enhances flexibility, allowing users to access any vehicle within a designated class, which is a feature essential for the dynamic nature of practical car-sharing. A formal security analysis proves our scheme is secure against both Type-I and Type-II adversaries under the random oracle model. Furthermore, performance evaluations demonstrate that our scheme achieves competitive efficiency while delivering significant practical functionalities. Future work will focus on extending this framework to multi-domain environments and developing practical cross-domain authentication protocols.

## Figures and Tables

**Table 1:** Comparative summary of the existing schemes.

Scheme	Technologies	Unforgeability	GA ^a^	DB ^b^
[33]	ECC, Short signature	Type II weak	✕	✕
[34]	ECC, Registration-based encryption	✔	✕	✕
[35]	ECC, Private key double insurance	Type I weak	✕	✕
[36]	ECC, Pseudonym mechanism, Low resource consumption	Type I weak	✕	✕
[41]	ECC, Password-based auth., Vehicle membership alteration	✔	✔	✕
[42]	Chebyshev chaotic map, Fuzzy verification, Honeywords	✔	✔	✕
[43]	Bilinear mapping, CMOS memristor-based auth.	✔	✔	✕
[44]	ECC, Fuzzy extractor, Biometric-based auth.	✔	✔	✕

✔: The scheme satisfies the feature. ✕: The scheme does not satisfy the feature. ^a^ GA: Granular authentication. ^b^ DB: Dynamic binding.

**Table 2:** Symbols and definitions.

Symbol	Definition
λ	Security parameter
TA	Trust Authority
KGC	Key generation center
${\mathrm{SV}}_{i}$	Sharing vehicle
${\mathrm{User}}_{j}$	User
*s*	Secret key of KGC
*t*	Secret key of TA
params	System parameters
${\mathrm{VID}}_{i}$	Identity of the vehicle
${\mathrm{PVID}}_{i}$	Pseudonym of the vehicle
${\mathrm{UID}}_{j}$	Identity of the user
${\mathrm{PID}}_{j}$	Pseudonym of the user
${\mathrm{PK}}_{i}$	Public key of the vehicle
$sk_{i}$	Private key of the vehicle
$d_{j}$	Private key of the user
$gid_{i},GID_{i}$	Category identifier
*z*	Group identifier
σ	Signature
$p_{j}$	Identifier update secret
$T_{\left\{i,j,z,l,m,r\right\}}$	Timestamp

**Table 3:** Execution time of cryptographic operations.

Symbol	Meaning	Time (ms)
$T_{bp}$	Bilinear Pairing	27.844
$T_{bpm}$	Pairing-based Scalar Multiplication	9.027
$T_{bpa}$	Pairing-based Point Addition	0.055
$T_{em}$	Scalar Multiplication	2.632
$T_{ea}$	Point Addition	0.018
$T_{htp}$	Hash to Point Operation	5.433
$T_{h}$	General Secure Hash	0.001

**Table 4:** Properties of the related schemes.

Scheme	Pairing-Free	Unforgeability	Revocation	User Decoupling	Category Range Selection
[48]	✕	✔	✕	✕	✕
[49]	✔	✔	✔	✕	✕
[50]	✔	✔	✔	✕	✕
[51]	✔	✔	✕	✕	✕
[52]	✔	✕	✕	✕	✕
[53]	✔	✕	✕	✕	✕
[54]	✔	✔	✕	✔	✕
Our scheme	✔	✔	✔	✔	✔

✔: The scheme satisfies the feature. ✕: The scheme does not satisfy the feature.

**Table 5:** Comprehensive overhead comparison with the related schemes.

Scheme	Computational Overhead		Communication Overhead (byte)
	Signature Generation (ms)	Signature Validation (ms)	
[48]	$5T_{bpm}+5T_{bpa}+2T_{htp}+2T_{h}=56.278$	$5T_{bp}+2T_{bpm}+2T_{bpa}+4T_{htp}+2T_{h}=179.118$	$|2G_{1}\left|+3\right|Z_{q}^{*}\left|+|T\right|$
[49]	$4T_{em}+2T_{ea}+4T_{h}=10.568$	$5T_{em}+3T_{ea}+4T_{h}=13.218$	$\left|G|+2\right|Z_{q}^{*}\left|+|T\right|$
[50]	$3T_{em}+T_{h}=7.897$	$5T_{em}+3T_{ea}+T_{h}=13.215$	$2|G|+2|Z_{q}^{*}\left|+|T\right|$
[51]	$T_{em}+2T_{h}=2.634$	$4T_{em}+3T_{ea}+3T_{h}=10.585$	$\left|G|+2\right|Z_{q}^{*}\left|+|T\right|$
[52]	$T_{em}+T_{h}=2.633$	$5T_{em}+3T_{ea}+2T_{h}=13.216$	$\left|G|+2\right|Z_{q}^{*}\left|+|T\right|$
[53]	$T_{em}+2T_{h}=2.634$	$4T_{em}+3T_{ea}+3T_{h}=10.585$	$\left|G|+2\right|Z_{q}^{*}\left|+|T\right|$
[54]	$2T_{em}+2T_{ea}+2T_{h}+T_{htp}=10.735$	$4T_{em}+3T_{ea}+2T_{h}+T_{htp}=16.017$	$2|G|+|Z_{q}^{*}\left|+|T\right|$
Our scheme	$T_{em}+2T_{h}=2.634$	$4T_{em}+4T_{ea}+4T_{h}=10.604$	$\left|G|+2\right|Z_{q}^{*}\left|+|T\right|$

**Table 6:** Comparison of the revocation feature.

Scheme	Support Revocation	Method	Overhead Complexity
[48]	✕	N/A	N/A
[49]	✔	Revocation list	Linear
[50]	✔	Blockchain database	Linear
[51]	✕	N/A	N/A
[52]	✔	Revocation list	Linear
[53]	✕	N/A	N/A
[54]	✕	N/A	N/A
Our scheme	✔	CRT	Constant

✔: The scheme satisfies the feature. ✕: The scheme does not satisfy the feature.

## Data Availability

Data are contained within the article.
